# A Competency-Based Approach to Expanding the Cancer Care Workforce Part III—Improving Cancer Pain and Palliative Care Competency

**DOI:** 10.1007/s13187-012-0354-z

**Published:** 2012-04-17

**Authors:** Kristen A. Cox, Alison P. Smith, Maureen Lichtveld

**Affiliations:** 1 C-Change, 1776 Eye Street, NW 9th Floor, Washington, DC 20006 USA; 2 Department of Global Environmental Health Sciences, Tulane University School of Public Health and Tropical Medicine, New Orleans, LA USA

**Keywords:** Pain, Palliative care, Competency, Cancer, Workforce shortage

## Abstract

As part of an effort to address shortages in the cancer workforce, C-Change developed competency standards and logic model-driven implementation tools for strengthening the cancer knowledge and skills of non-oncology health professionals. These standards and tools were applied by four diverse grant programs to yield gains in the management of pain and palliative care, thereby improving the quality of care for individuals experiencing or recovering from cancer treatment. The results from the four grant sites and tools used to achieve them are described in this article.

## A Workforce Shortage across All Cancer Health Professions

Our Nation’s capacity to fight cancer and improve the quality of care for patients depends on the quality and quantity of the cancer workforce—the number of health professionals and the adequacy of their training. Nearly all of the professional disciplines who play a role in the delivery of comprehensive cancer services are experiencing a shortage including physicians, nurses, social workers, pharmacists, public health workers, researchers, technologists, and cancer registrars. The rising incidence of cancer, an aging population, and an increase in cancer survivorship all predict an increased demand for health services. These trends threaten our ability to provide timely and comprehensive cancer care.

The supply of cancer-related research, clinical, and public health professions is inadequate and is projected to worsen among nurses, social workers, oncologists, radiation oncologists, pharmacists, researchers/scientists, and imaging technologists while the current and projected demand for cancer care is expected to increase. This imbalance between supply and demand is quantitatively exemplified below:

- The current physician shortage is approximately 8 % and is projected to be greater than 20 % in 2025 [3]
- The supply of oncologists is only predicted to increase by 14 % by 2020, creating a shortage of 2,500 to 4,080 oncologists [4]
- More than 20 % of the US population lives in areas deemed by the federal government as health professions shortage areas without access to adequate medical care [5]
- One third of critical access hospitals lack a surgeon living in the county [6]
- By 2020, the shortage of registered nurses will be greater than 1 million [7]
- Only 33,000 of 120,000 registered nurses who specialize in oncology are certified [5, 8]
- The social work labor force is older than most professions, with nearly 30 % of licensed social workers over age 55 [9]
- Currently, the U.S. has approximately 4,400 hospice and palliative medicine physicians, but 6,000 to 18,000 are needed to meet the gap between supply and demand [10]
- By 2015, 81,000 additional clinical laboratory technologists will be needed to replace retiring staff and another 68,000 to fill newly created positions [11]
- The projected shortage of more public health workers by 2020 is 250,000 [12]
- While 25 % of the U.S. population is comprised of African Americans, Hispanics, and Native Americans, medical training programs are comprised of fewer than 7 % of underrepresented minorities [13]

## Unique Organization with a Unique Approach

Founded in 1998, C-Change is the only organization that assembles the Nation’s cancer leaders from the three sectors—private, public, and not-for-profit—and from across the cancer continuum—prevention, early detection, treatment, to palliative care and survivorship. The mission of C-Change is to eliminate cancer at the earliest time possible by leveraging the expertise and resources of its members. The cancer workforce shortage is one challenge that C-Change’s members identified as an important priority for collaborative action. In an effort to address this shortage, the C-Change Cancer Core Competency Initiative was developed to equip non-oncology professionals with the knowledge and skills to better meet the needs of an aging and increasingly diverse population of people at risk for or living with cancer.

Convened by C-Change, a national, multi-disciplinary panel of leaders and experts developed competency standards that define the knowledge and skills needed by all healthcare providers. The competency standards can be applied in any healthcare setting, by any health discipline, and address issues spanning the continuum of care, cancer science, and care coordination and communication. Implementation tools include a logic model and curriculum validation template. Defining core competencies is a widely recognized approach to developing and maintaining key knowledge and skills in the workforce, and is one important approach toward expanding the cancer workforce.

As discussed in previously published work, the competency standards, coupled with a model for planning, implementation, and evaluation were successfully pilot-tested in four diverse healthcare settings targeting different professional populations and cancer topics [1, 2]. The program participants achieved quantitative and qualitative improvements in their knowledge, skills, and attitudes. Similarly, the host organizations benefited from the experience, and in each instance developed tailored strategies to build on the accomplishments of the pilot projects. All sites reported that the competency tools demonstrated both flexibility and utility to meet the goals of their individual organizations. A full description of the standards, tools, and pilot site results can be found at www.cancercorecompetency.org.

## Pain and Palliative Care Core Competency Grant Program

Building on the pilot grants, C-Change awarded four additional cancer core competency grants that were focused on identifying, treating, and managing cancer pain, and the benefits of palliative care. Cancer pain and symptoms can manifest in many different forms, and can also be a telling indication that cancer has returned, or that the care an individual is receiving or being offered is not sufficient to manage the effects of the disease or the side effects of treatment. Research shows that 26 % of unscheduled hospital admissions are due to uncontrolled pain [14], and nearly 40 % of 5-year cancer survivors and 60–85 % of people with advanced cancer report pain [15]. Care that is intended to enhance quality of life should be a priority throughout the disease trajectory [16], yet current data show that palliative care is being delivered too late in the course of disease, often too late to adequately alter the quality and delivery of care that is provided to patients with cancer [17].

To adequately address cancer pain and accompanying psycho-social aspects of cancer, the entire healthcare workforce should ideally be armed with the knowledge and skills necessary to identify, treat, and refer patients for appropriate pain and palliative care services. In July of 2009, C-Change invited any healthcare organization, association, or comprehensive care control coalition to apply for a grant aimed at strengthening the cancer pain and palliative care knowledge, skills, and attitudes of non-oncology health professionals. C-Change convened an Advisory Committee of pain and palliative care experts, received 18 proposals, and awarded grants to four sites to develop competency-based pain and palliative care programs. The programs were developed and implemented based upon the below pain and palliative care competency standards to improve the pain management knowledge, skills, and attitudes among non-oncology health professionals:

### Palliative and End of Life Competencies

1. General
  - Define palliative and end of life care.
  - Assess that resources for palliative and end of life care and insurance coverage are consistent with current recommendations.
  - Refer patients to community palliative and end of life care and support resources.
  - Explain the role of hospice care.
  - Manage symptoms of the cancer patient.
  - Incorporate end of life comfort strategies for the dying cancer patient.
2. Pain management
  - Describe the methods used to diagnosis pain throughout the progression of the disease.
  - Differentiate between acute and chronic pain symptoms.
  - Describe the characteristics used to assess pain: frequency, intensity, and site.
  - Perform a pain assessment.
  - Explain the different treatment options for pain.
  - Perform a pain-related history taken during a physical examination.
  - Manage pain and analgesic side effects.

When applying the pain and/or palliative care competencies, applicants were encouraged to address one or more contemporary pain and palliative care issues:

- Focus on professionals who assess, prescribe, and manage care for pain and palliation (MD, DO, PA, NP, RN, Pharmacist)
- Promote awareness of a full range of treatment modalities including drugs, devices, and therapies
- Address culture-specific pain management issues and disparities
- Embrace a multi-disciplinary approach and systems that drive assessment and reassessment
- Recognize pain management needs in inpatient and outpatient settings, medical home models encouraged
- Address professional practice drivers such as prescribing in the context of the United States Drug Enforcement Agency surveillance
- Address patient-specific insurance policy issues that may impact benefit coverage such as pain classification and documentation
- Promote patient education and self advocacy

Grant sites were required to develop programs that could measurably improve the patient assessment, treatment, monitoring, and outcomes related to pain and palliative care in the context of cancer; refine program plans based upon the planning and implementation phases of the grant program; disseminate the results; and contribute to C-Change’s online public repository of planning documents, evaluation tools, and lessons learned from various sites.

## Grant Site Expectations

Selected grant sites were expected to work in collaboration with the C-Change Advisory Committee and staff to:

- Participate in a 1–2 day project orientation session with the expert Advisory Committee to refine and focus their program plans
- Collaborate on conference calls with C-Change and other grant sites during the planning process
- Refine and expand the pain competency definitions as needed
- Form a site-specific planning committee with the expertise to support the development of curriculum materials, instructional methods, and evaluation tools
- Identify a target professional population and competency topic guided by the Core Competency Definitions
- Complete a needs assessment with the target professional population
- Utilize C-Change logic model and curriculum validation templates for program design
- Fulfill the IRB requirements of participating institution if appropriate
- Implement a program plan within the target professional population
- Assure a minimum level of participant accrual
- Evaluate program impact on learner knowledge, skills, and attitudes regarding the cancer competency topic
- Record and share all project data, methods, teaching and communication tools
- Publish a final report
- Write and submit an article for publication in a relevant professional journal
- Submit a proposal to present program findings at a professional conference
- Serve as a program reference for other sites

## Scope of Four Unique Cancer Core Competency Programs

Four of the eighteen applicants were awarded grants: Iowa Cancer Coalition (ICC), University of Florida (UF), Virginia Commonwealth University (VCU), and South Puget Intertribal Planning Agency (SPIPA). Each grant site applied the Cancer Core Competency tools and methods to address cancer topics that specifically met the unique needs of the populations they served, ultimately enhancing the knowledge, attitude, and skills of the targeted health professionals (Table 1). Through the use of logic models, validation templates, and guidance of the C-Change advisory committee, each program was developed to ensure that the desired improvements in knowledge, skills, and attitudes were measurable and sustainable.

**Table 1:** Overview of pain and palliative care cancer core competency programs

	Iowa Cancer Coalition (ICC)	University of Florida (UF)	Virginia Commonwealth University (VCU)	South Puget Intertribal Planning Agency (SPIPA)
Cancer Topic	Describe palliative and end of life care Explain the role of hospice	Pain and cancer-related symptoms and management resources	Pain management in pediatric patients	Culture-specific cancer pain

Healthcare discipline/ Learner Target Audience	Nurses, Medical Assistants	Physicians, Nurses, Social Workers, Office Staff	Medical Students, Pediatric Residents	Native Health Workers, Caregivers

Type / Level of education and experience	Practicing professionals; AD, BSN, Certificate	Practicing professionals, MD, RN, MSW, Diploma	Students, Pre-Professional	Variable education and training as “lay” community health worker

Practice Setting	Rural long term care facilities	Rural health, primary care clinics (mostly Federally Qualified Health Centers)	Pediatric Clinic and Medical Center	Native American communities

Each grant site developed unique tools to teach, apply, and sustain the learning process. Teaching methods included didactic lectures, video patient scenarios, and an interactive online learning program. Tools for applying and sustaining improved knowledge and skills included standard order sets, scripts for difficult conversations, patient-symptom journals, and culturally appropriate discomfort barometers in place of traditional pain scales.

## Highlights of Program Results

Each program targeted a specific professional population, defined program goals, developed a unique approach, and achieved measureable results.

## Iowa Cancer Coalition—End of Life Communication and Collaboration

The Iowa Cancer Consortium (ICC) is dedicated to reducing the burden of cancer in Iowa through priorities set in its State Cancer Plan including the need to improving pain and palliative care and strengthening the cancer workforce. Over 50,000 Iowans live in 840 long-term care facilities, which are often care providers at the end of life. The ICC grant program targeted staff working in long-term care facilities in northeastern Iowa including nurses, social workers, and medical assistants. The educational conference addressed the role of palliative care and hospice, approaches to difficult conversations, and care management techniques. (Table 2)

**Table 2:** Iowa Cancer Coalition—End of Life Communication and Collaboration

Population	▪ Nurses and medical assistants practicing in rural, long-term care facilities

Program goals	▪ Describe palliative and end of life care
	▪ Explain the role of hospice

Unique approach	▪ Use of order sets for palliative/ hospice care
	▪ Use of scripts/ talking points for difficult conversations

Results at completion of grant cycle	▪ 40 participants from 22 communities in northeast Iowa
	▪ 12 % increase in knowledge from pre-to post-test scores,
	▪ Possible addition to Iowa College of Nursing distance learning cancer module
	▪ Creation of state-wide resources for pain and palliative care services

## University of Florida—Pain and Palliative Care Competency Training for Non-Oncology Health Professionals Working in Rural Settings

The University of Florida program was a collaborative project with three organizational partners: The University of Florida (UF) Department of Community Health & Family Medicine and the UF-Shands Cancer Center; and the Rural Health Partnership of North Central Florida, Inc. (RHP); all three partners serving residents from nine predominantly rural and underserved counties. This population has the highest cancer mortality rates in the state of Florida, and as a result of the rural setting, these communities are often underserved due to a low concentration of primary care and oncology-specialized healthcare providers and services. Rural health facilities depend on the knowledge and skills of health professionals in outpatient primary care settings to address cancer-related pain management and palliative care, areas where primary care professionals are often under trained. (Table 3)

**Table 3:** University of Florida—Pain and Palliative Care Competency Training for Non-Oncology Health Professionals Working in Rural Settings

Population	▪ Rural primary care physicians, nurses, social workers, and office staff

Program goals	▪ Improve knowledge, skills and confidence in describing cancer-related symptoms
	▪ Improve methods to screen for health care and services needs
	▪ Strengthen referral pathways and palliative care resources for patients

Unique approach	▪ Multidisciplinary and multimedia program instructed by an oncologist and an oncology social worker;
	▪ Use of videos with patient perspectives
	▪ Use of video with a standardized patient

Results at completion of grant cycle	▪ 120 participants
	▪ 21 % increase in overall level of confidence
	▪ 90 % reported improvement in gaining new knowledge and skills to provide better patient care

## Virginia Commonwealth University—Pediatric Pain Management: The Development of an Online Competency Module

Virginia Commonwealth University (VCU) Medical Center is a comprehensive academic medical center in central Virginia that conducts basic, translational, and clinical cancer research, provides state-of-the-art treatments and clinical trials, promotes cancer prevention and education, and has been designated by the Robert Woods Johnson Foundation as a Palliative Care Leadership Center. Despite advances in pain research and improvements in practice, VCU recognized the continued lack of training resources for students and practitioners to specifically improve pediatric pain management. Their grant program aimed to expand the existing *VCU Pain Management: An Online Curriculum* with a Pediatric Pain Management module. (Table 4)

**Table 4:** Virginia Commonwealth University—Pediatric Pain Management: The Development of an Online Competency Module

Population	▪ Third and fourth year medical students, pediatric residents at VCU

Program goals	▪ Describe the pathophysiology of pain in children
	▪ Recognize the barriers to effective pediatric pain management
	▪ Perform a pediatric pain assessment
	▪ Manage pediatric-related pain and analgesic side effects

Unique approach	▪ Online, interactive course
	▪ Course available online as national resource

Results at completion of grant cycle	▪ 302 participants to date
	▪ 28 % increase in knowledge
	▪ 331 % increase in confidence in assessing pain in pediatric patients^1^
	▪ 403 % increase in confidence in treating pain in pediatric patients^1^
	▪ 255 % increase in confidence in ability to prescribe opioids to treat pain in pediatric patients^1^

^1^Percentage change = [(Post test score—Pretest score) / Pretest score] × 100. So, if a participant scored 25 points out of 100 possible points on the pretest and scored 100 points out of 100 possible points on the post test, then the percentage change would be [(100–25)/25)] × 100 = 300 %

## South Puget Intertribal Planning Agency—Addressing Culture-Specific Pain Management: Creating a Common Ground between Community Members and Caregivers to Address Native American Cancer Pain and Palliative Care

The South Puget Intertribal Planning Agency’s (SPIPA) Comprehensive Cancer Control Program serves five Tribes (Chehalis, Nisqually, Shoalwater Bay, Skokomish, Squaxin Island), providing services to over 13,000 Native Americans located in southwestern Washington. Tribal communities served by SPIPA cope with barriers in effective pain management, often associated with cultural, religious, and traditional tribal beliefs, not always compatible with western medical practices, and compounded by a lack of awareness and access to services. To address these issues, SPIPA conducted assessments of elders in the five tribes to develop culturally appropriate trainings for Tribal members on “Recognizing and Documenting Cancer Related Distress and Discomfort.” The programs aimed to strengthen cancer patient and caregiver knowledge, skills, and attitudes regarding emotional distress and physical discomfort, and offered ways to better communicate with caregivers and health practitioners. (Table 5)

**Table 5:** South Puget Intertribal Planning Agency—Addressing Culture-Specific Pain Management: Creating a Common Ground Between Community Members and Caregivers to Address Native American Cancer Pain and Palliative Care

Population	▪ Native health workers, cancer survivors, and caregivers from the five tribes served by SPIPA

Program goals	▪ Address culture-specific cancer pain
	▪ Explain how cancer pain differs from other types of pain
	▪ Perform a cancer pain assessment
	▪ Differentiate between physical discomfort and emotional distress within the context of historical pain

Unique approach	▪ Thorough pre-assessment with tribal elders were critical in developing program content relevant to this population
	▪ Developed a patient symptom journal
	▪ Developed a culturally appropriate “Discomfort” Barometer

Results at completion of grant cycle	▪ 102 participants
	▪ 120 % improvement in confidence to identify and report symptoms

## Lessons Learned

Grant recipients experienced the benefits of a competency-based approach for developing sustainable education programs. The structured, yet flexible approach allowed each site to choose a competency and adapt it to the target audience, allowing individual learners to gain knowledge and skills required to optimize their role in cancer care delivery. Using a competency -based structure forced the program developers to link each learning activity to a measurable outcome that would ultimately improve cancer care in the population they serve.

Competency-based programs require significant planning, an investment that is greater than a typical 1-hour continuing education program. (Table 4) While this initially seemed to take more time and energy than the grant recipients anticipated, each site came to the realization that the investment was necessary to achieve measurable and lasting change in their professional population and health system.

Voluntary learning opportunities compete with the day-to-day priorities of work and family commitments and must overcome many barriers to fully engage the learners. To foster participation, many grantees provided incentives such as gasoline fuel cards and meals as an additional impetus for participation.

An unintended benefit noted by the grant sites was the power of collaboration and the benefits of leveraging individual assets for the common good. Bringing together different partners not only enriched the program itself, but also strengthened relationships in the community, creating partnerships which exist beyond the completion of the programs.

## Call for Further Application of C-Change Cancer Core Competency Initiative

**Fig. 1:**
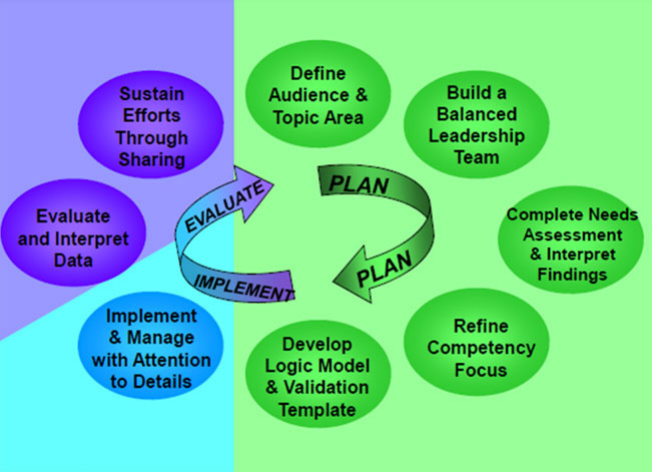
Steps for program development

As demonstrated above and in previously published sources, the C-Chance Cancer Core Competency Initiative can be applied in any setting, focused on any level of learner, and in any health discipline to improve the competency of the non-oncology workforce. C-Change encourages individuals to access the detailed grant site reports for further information on the programs, and to access the tools and methods when developing a cancer core competency program aimed at improving cancer care. To effectively address the projected demand for cancer care, sustained interventions are needed to recruit, retain, and retrain health professionals. The Cancer Core Competency Initiative represents an effective strategy to better leverage the existing health workforce serving the increasing health care- and services needs of people at risk for and living with cancer (Fig. 1).
